# Arc-Welding Spectroscopic Monitoring based on Feature Selection and Neural Networks

**DOI:** 10.3390/s8106496

**Published:** 2008-10-21

**Authors:** P. Beatriz Garcia-Allende, Jesus Mirapeix, Olga M. Conde, Adolfo Cobo, Jose M. Lopez-Higuera

**Affiliations:** Photonics Engineering Group, University of Cantabria, Avda. de los Castros S/N, 39005 Santander, Spain; E-Mails: jesus.mirapeix@unican.es; olga.conde@unican.es; adolfo.cobo@unican.es; miguel.lopezhiguera@unican.es

**Keywords:** Arc-welding, fiber sensor, spectral processing, plasma spectroscopy, on-line monitoring

## Abstract

A new spectral processing technique designed for application in the on-line detection and classification of arc-welding defects is presented in this paper. A non-invasive fiber sensor embedded within a TIG torch collects the plasma radiation originated during the welding process. The spectral information is then processed in two consecutive stages. A compression algorithm is first applied to the data, allowing real-time analysis. The selected spectral bands are then used to feed a classification algorithm, which will be demonstrated to provide an efficient weld defect detection and classification. The results obtained with the proposed technique are compared to a similar processing scheme presented in previous works, giving rise to an improvement in the performance of the monitoring system.

## 1. Introduction

Arc welding on-line monitoring has been an active research topic in the last decades. The complexity of the physics involved in this process [1], the difficulty to find models with computational times allowing real-time analysis, and their increasing relevance in several industrial niches have prompted the search for reliable procedures to ensure the quality of the resulting welds. The use of non-destructive evaluation techniques (NDT) such as penetrant liquids, X-rays, magnetic particles or infrared thermography [2], is typically combined with procedure trials in test specimens or coupons, in an attempt to establish optimal values for the input welding parameters (welding current, shielding gas flow rate, separation between the electrode tip and the seam). On-line monitoring systems have been proposed for arc welding processes [3-5]. In this regard, plasma optical spectroscopy is a promising alternative, as there exists a correlation between some plasma spectroscopic parameters, e.g. the plasma electronic temperature, and the appearance of defects in the seams. In addition, on-line monitoring by means of real-time analysis is feasible with this approach [6-7].

In terms of optical sensor system proposals, the particular characteristics of the arc-welding process have made it difficult to find a non-invasive solution. Input optics based on collimators attached to the welding torch have been proposed [8], but these have limited applicability in general terms. In a previous communication we proposed a new sensor arrangement based on the use of an optical fiber embedded within a TIG torch [9]. In spite of the aggressive environment to be found in the vicinity of the plasma column, we demonstrated the feasibility of the proposed sensor system to be applied for on-line arc-welding quality monitoring.

When the traditional spectroscopic approach for welding quality monitoring is chosen, plasma electronic temperature profiles are obtained by using two or more atomic emission lines within those contributing to the plasma. Defect detection can be accomplished with this approach, but defect classification has not yet been efficiently achieved. A new processing technique based on the use of PCA (Principal Component Analysis) as the dimensional reduction stage, and an ANN (Artificial Neural Network) designed and trained to perform the defect classification was proposed in a previous paper [10]. Although the validity of the technique was demonstrated, it gave rise to further investigations, mainly focused on providing information regarding the spectral bands selected by PCA to be used as the ANN inputs.

Some of the disadvantages found in the traditional feature extraction techniques such as Principal Component Analysis (PCA) or Linear Discriminant Analysis (LDA) are listed in the following [11]:

- When analyzing an unknown spectrum it is necessary to measure all the original features or spectral bands in order to perform the compression prior to its classification.
- The interpretation of the results becomes a complex task given that the obtained features can not be associated with any of the spectral bands of the compounds under test.
- The obtained results can not be extended to other classifiers.

In this paper an algorithm based on feature selection, the Sequential Floating Forward Selection (SFFS) [12] is proposed to obtain the main features or spectral bands to be used afterwards in the classification stage of spectral data from an optical fiber-based sensor system. SFFS solves the disadvantages of PCA and LDA mentioned above, and in the particular case of plasma spectrum analysis, allows to establish an association between the selected bands and their corresponding emission lines found in the plasma spectra. A properly trained Artificial Neural Network (ANN) will be employed in the subsequent classification stage.

## 2. Plasma optical spectroscopy

As mentioned above, plasma optical spectroscopy is regarded as a promising technique for the on-line welding quality monitoring problem. This approach is typically based on the estimation of the plasma electronic temperature *T_e_* by means of the following simplified expression [13]:

(1) $$T_{e}=\frac{E_{m}\left(2\right)-E_{m}\left(1\right)}{k\ln\;\left[\frac{E_{m}\left(1\right)I\left(1\right)A\left(2\right)g_{m}\left(2\right)\;\lambda \left(1\right)}{E_{m}\left(2\right)I\left(2\right)A\left(1\right)g_{m}\left(1\right)\;\lambda \left(2\right)}\right]}$$

where *E_m_* is the upper level energy, *g_m_* the statistical weight, *A* the transition probability, *λ* the wavelength, *I* the emission line relative intensity and *k* the Boltzmann constant. It can be seen that Equation (1) implies the utilization of two emission lines (of the same element in the same ionization stage). Although the temperature obtained with this approach may imply an error in its mean value, the main interest lies in the localization of strong perturbations in the *T_e_* signal, which will be associated with weld defects.

Equation (1) is a simplification of the so-called Boltzmann-plot (derived from the Boltzmann equation [14]), which allows the use of several emission lines for the *T_e_* calculation process:

(2) $$\ln\left(\frac{I_{mn}\;\lambda_{mn}}{A_{mn}\;g_{m}}\right)=1\text{n}\left(\frac{\text{hcN}}{Z}\right)-\frac{E_{m}}{kT_{e}}$$

where *h* is the Planck's constant, *c* is the light velocity, *N* the population density of the state *m* and *Z* the partition function. The representation of the left-hand side of Equation (2) versus *E_m_* has a slope inversely proportional to *T_e_*.

Although the exactitude of the estimated temperatures will depend on the selected emission lines, Equation (2) provides less noisy temperature profiles, which is of interest to avoid false alarms or the lack of detection of existing defects. However, Equation (2) tends to be avoided for on-line welding monitoring given its higher computational cost.

Although there are several proposals based on plasma optical spectroscopy [7-8, 15], the interpretation of the resulting temperature profiles is not always easy, and an efficient algorithm allowing defect classification is still to be formulated. Moreover, the identification and selection of the emission lines participating in the analysis could lead to ambiguities, especially if the system is designed to be fully-automated.

Within this framework, the proposal of solutions using ANNs seems natural [16-17], as they could provide a computationally efficient system and, in addition, a possible classification of the weld defects. The latter could be useful if a real-time feedback on the process is planned to try to avoid the appearance of flaws. On the other hand, given that there is redundancy in the plasma spectral data, an initial stage offering data compression will be suitable.

## 3. Data analysis

### 3.1. Sequential Floating Forward Selection

Data compression by means of the SFFS algorithm is performed in two stages: redundancy reduction and spectral band selection.

#### 1. Redundancy reduction

Spectral bands providing the same information to discriminate between correct welds and the different kind of defects have to be determined. A high correlation coefficient between two bands, nearly 1, means that both offer the same information. Therefore, a data decorrelation is performed. First of all the correlation coefficient matrix is calculated and 51 blocks of correlated spectral bands, i.e. with a correlation coefficient higher than 0.99, are obtained. Afterwards, a spectral band of each block, the most discriminant one according to the Bhattacharyya distance is selected. The Bhattacharyya probabilistic distance between two normal distributions is:

(3) $$J_{B}=\frac{1}{4}{\left(\mu_{2}-\mu_{1}\right)}^{T}\left[\sum_{1}+\sum_{2}\right]\left(\mu_{2}-\mu_{1}\right)+\frac{1}{2}\log\left(\frac{\left|\sum_{1}+\sum_{2}\right|}{2{\left(\left|\sum_{1}\right|\cdot \left|\sum_{2}\right|\right)}^{\frac{1}{2}}}\right)$$

where *μ_i_* is the mean of the *i* class; Σ*_i_* its covariance matrix and |*A*| stands for the determinant of matrix *A*. In this case three different instabilities are discriminated from correct welds, therefore the criterion to select the most discriminant band must include the Bhattacharyya distance between every two distributions:

(4) $$J=\sum_{i=1}^{4}\sum_{j=i+1}^{4}P_{i}P_{j}J_{ij}$$

where *P_i_* is the occurrence probability of correct welds and each of the three instabilities respectively.

#### 2. Feature Selection

The problem of feature selection consists in, given a set of candidate features, selecting a subset with the best performance under some classification system. In this particular case the system performing the classification task is an Artificial Neural Network and the features are the different wavelengths of the acquired plasma spectra.

The SFFS algorithm begins with an empty feature subset. It iteratively adds and removes features until some termination criterion is fulfilled. This criterion is established by the user and it is the maximum number of features to be used. The term “forward” is used because it begins with an empty feature subset, and “floating” because after each forward step (addition of a new spectral band) some backward steps (removal of several spectral bands) are applied as long as the resulting subsets are better than the previously evaluated ones. The selection criterion is an objective function evaluated for each selected feature subset and it is a measurement of the separability of the classes. The probabilistic Bhattacharyya distance for a multiclass discrimination is again applied to measure this separability.

**Figure 1.**
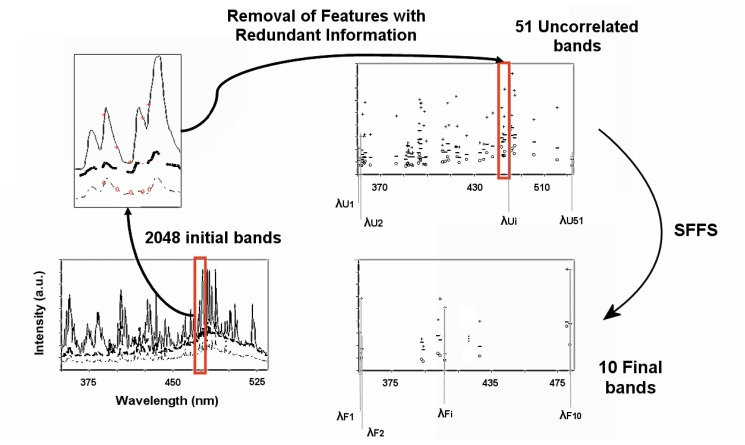
Scheme of the spectral band compression and selection by means of SFFS.

The spectral dimensionality reduction procedure is summarized in Figure 1.

### 3.2. Clasification

The final selected bands are then classified using an artificial neural network [18-19]. The net is a multilayer feed-forward network, where a Back-propagation learning algorithm has been employed. It consists of an input layer and a single hidden layer, the two of them with 20 neurons or processing elements, and an output layer with only four neurons. The first neuron of the output layer is activated, which means that its output is nearly 1, when the spectrum under test is classified as a correct weld and the second one if a reduction in the gas flow is found. The activation of the third neuron indicates the presence of an incision (perturbation in the piece thickness) and finally the fourth neuron is activated in case of an insufficient welding current. The neurons in all layers have a log-sigmoid transfer function. This network topology is simpler than the one used in the work previously mentioned [10], then another additional advantage, a reduction in the mean classification time, is provided by the employment of SFFS instead of PCA in the compression stage. In the following Section it will be demonstrated that this enhancement in the computational performance causes an inappreciable decrease in the system performance concerning the detection of instabilities.

## 4. Experimental issues

**Figure 2.**
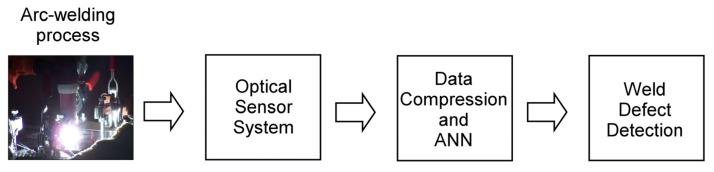
Block diagram of the on-line welding quality monitoring system.

Several bead-on-plate welding tests were carried out to check the proposed analysis technique. A TIG arc-welding system, composed of a Kemppi Mastertig 220 power supply and a Kemppi TIG torch TTC 220 using tungsten electrodes (1 mm diameter) were employed for the welding tests. The distance between the electrode tip and the plates was approximately 2 mm, and the electrode tip preparation was conical (1 mm length). The extension of the electrode from the exit of the shielding cup (8 mm diameter) was approximately 3 mm, and welding currents from 30 to 50 A were chosen. The specimens used in the experiments were stainless-steel AISI 304 plates attached to a positioning system (Newport MM4005 controller and MTM100PP1 positioning stage (1 μm precision)). Argon was selected as shielding gas, with a constant flow rate of 12 L/min. As mentioned in Section 1, an optical fiber (Ocean Optics P50-UVVIS) was used as a spectroscopic sensor embedded within the TIG torch [10]. The use of one of the gas nozzle exits to guide the fiber tip provides a cooling effect by means of the shielding gas. In addition, the shielding gas flow also prevents projections from the welding pool from damaging the fiber sensor. More than 200 welding tests have been carried out with the proposed optical sensor arrangement, enabling a complete spectroscopic analysis. A schematic representation of the complete monitoring system, including the optical fiber sensor, is presented in Figure 2.

**Figure 3.**
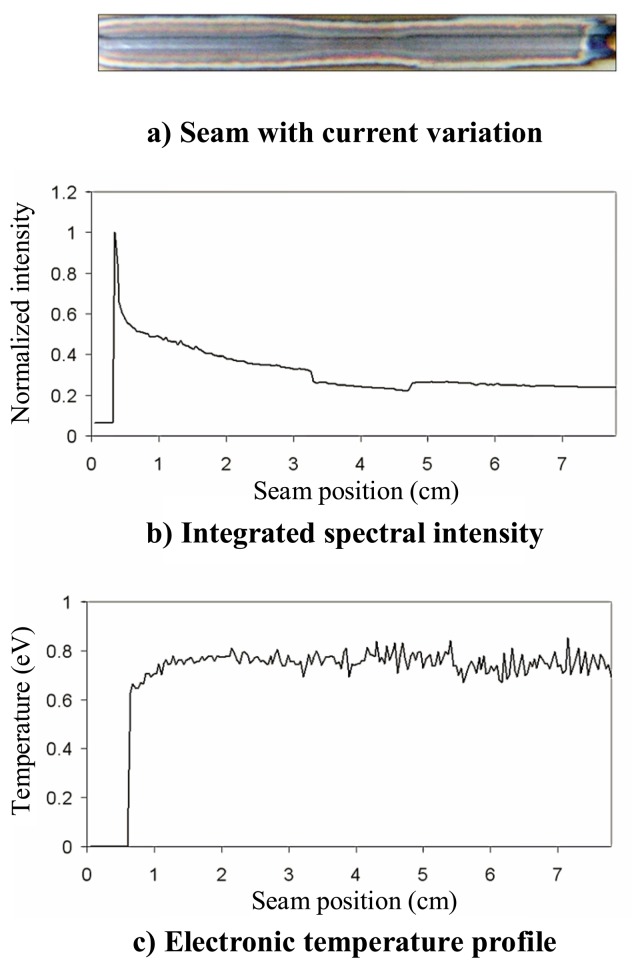
Defective weld and associated electronic temperature profile.

A defective seam is presented in Figure 3(a), where a reduction in the welding current (50 to 44 A) can be appreciated from *x* ≈ *3.5* to *x* ≈ *5* cm.

An initial analysis was carried out by estimating the plasma electronic temperature using Equation (2) with three Ar II lines (with central wavelengths located at approximately 461, 480.5 and 485 nm). As can be appreciated in Figure 3(c), the slight variation in the welding current is not associated with any clear perturbation in the temperature profile, whose mean value is 0.76 eV. As an additional analysis, the integrated spectral intensity for each spectral capture is also shown in Figure 3(b), where, as expected, there is a subtle perturbation between *x* ≈ *3.5* and *x* ≈ *5* cm.

**Figure 4.**
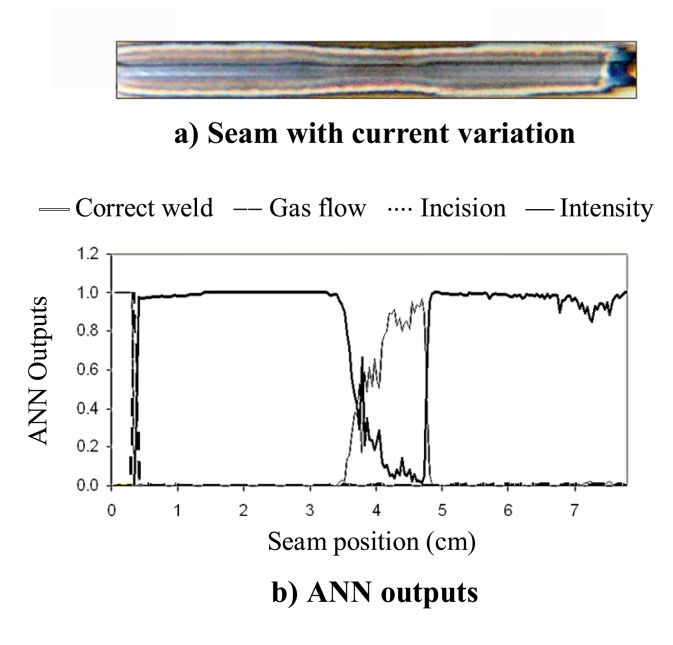
Defective weld and associated ANN outputs.

The result provided by the combination of SFFS and the designed ANN applied to the previous seam is depicted in Figure 4. It can be seen in Figure 4(b) that the ANN output associated with the detection of low current is activated, what implies a classification of the flaw, thus providing more information regarding the process. As expected, when the “low current” output is activated, the output labeled as “correct weld” exhibits values lying under the selected threshold (0.5).

**Figure 5.**
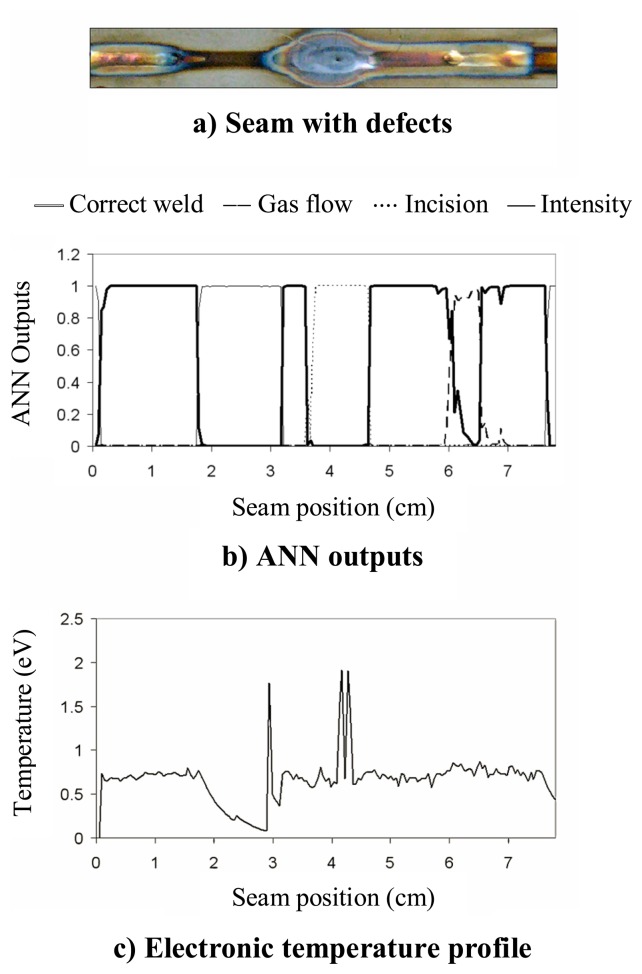
Defective weld and associated ANN outputs and *T_e_* profile.

Another defective weld is presented in Figure 5, where different defects can be found: a reduction in the welding current (50 to 30 A) from *x* ≈ *1.8* to *x* ≈ *3.2* cm, an incision in the plate width (orthogonal to the weld direction) from *x* ≈ *3.7* to *x* ≈ *4.6* cm and a perturbation in the shielding gas flow from *x* ≈ *6* to *x* ≈ *6.5* cm. The resulting ANN outputs are depicted in Figure 5(b), showing a good correlation with the events mentioned above. It is worth noting that the *T_e_* profile, presented in Figure 5(c), would allow to identify the first two defects to be found in the seam: the ones provoked by the variation in the weld current and the plate incision. However, there is no clear perturbation in the temperature signal associated with the shielding gas flow perturbation. *T_e_* was again estimated with the same Ar II emission lines used in Figure 3(c).

Given that the number of spectral bands selected by SFFS is a key parameter in terms of the computational efficiency of the proposed solution, the effect of the suppression of some of these bands on the ANN classification error has been studied. A first step performed was the classification of the spectral bands chosen by SFFS in terms of their variability [20] (Table 1). The variability measure for a given component (spectral band) is defined as the quotient between the deviation of the class-means and the mean of the deviation of each class. The numerator represents how much the classes are separated, therefore it will be preferred to be large. On the contrary, the denominator expresses the compactness of the classes, and small values would be desired.

**Figure 6.**
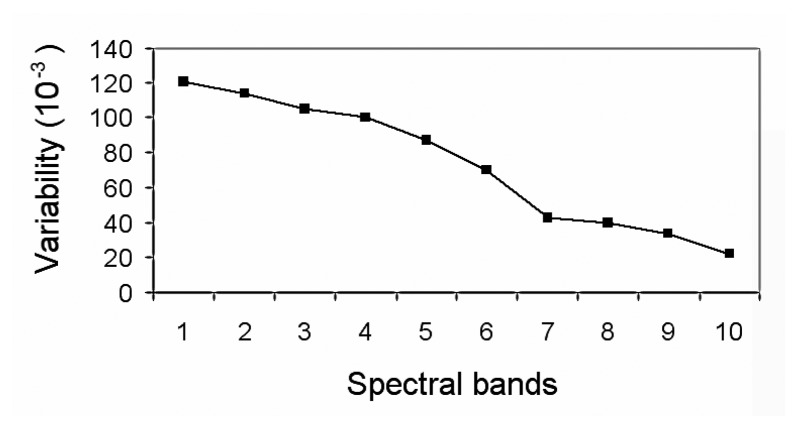
Spectral bands classified in terms of their variability.

In Table 1*Band N*° is the label associated with each spectral band, λ is the central wavelength of each emission line, and *Emission Line* provides the identification for each chosen spectral band. The evolution of the variability for the selected bands is presented in Figure 6.

**Figure 7.**
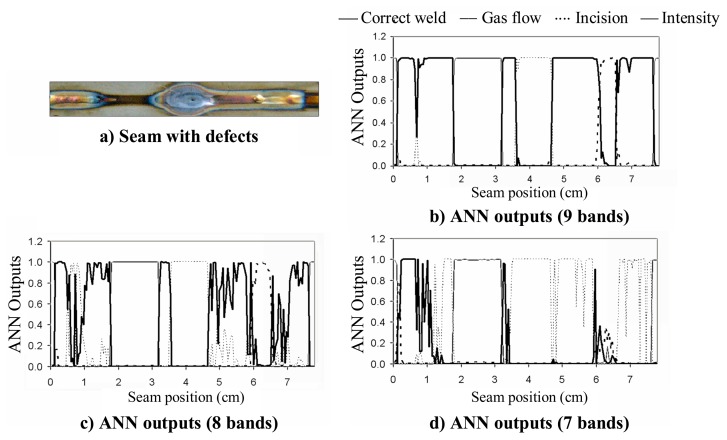
Study on the effect of the number of selected spectral bands on the ANN outputs.

An interesting study is shown in Figure 7, where it has been analyzed the effect on the ANN outputs of reducing the initial set of selected spectral bands.

The same seam analyzed in Figure 5 is considered again for this study. In Figure 7(b) it can be observed that when only nine spectral bands are chosen in terms of their variability, the result provided by the ANN is quite similar to the one presented in Figure 5(b). However, if a further reduction is performed, and only eight spectral bands are taken into account [Figure 7(c)], the ANN outputs clearly exhibit less correlation to the expected results. This effect can be also appreciated in Figure 7(d), where only seven bands were chosen.

## 5. Conclusions

A technique based on feature selection to reduce spectral dimensionality of data from an embedded fiber-sensor has been designed and successfully validated in the on-line detection and classification of arc-welding defects. Compared to feature extraction based-on methods, its main advantage lies in the identification of the physical meaning of the selected spectral bands. In addition, in this particular application, a reduction in the complexity of the classification algorithm has been achieved, what also implies an enhancement in the computational performance of the proposed technique. Finally, studies regarding the variability of the spectral bands selected by SFFS and their influence in the resulting ANN outputs have been also carried out, showing that even with just 9 bands the proposed technique will be feasible. An experimental validation of the approach has been performed by means of TIG welding tests in the laboratory. The solution proposed in this paper is intended to be applied in a real industrial scenario.

## Figures and Tables

**Table 1.** Variability for the selected emission lines.

**Band N°**	**λ (nm)**	**Variability**	**Emission Line**
1	407.22	1.21E-02	Fe I
2	404.30	1.14E-02	Mn I
3	482.43	1.05E-02	Mn I
4	356.92	1.00E-02	Ni I
5	402.84	8.73E-03	Mn I
6	428.09	7.00E-03	Ar II
7	356.08	4.24E-03	Ar II
8	394.02	4.01E-03	Cr I
9	393.20	3.38E-03	Fe I
10	480.63	2.22E-03	Ar II
